# A Bribe

**Published:** 1860-07

**Authors:** 


*A Bribe.*—A "Homœopath" writes to the morning papers stating that in answer to the appeals of various hospitals for further funds, he has addressed an offer to St. Mary's, St. George's, Middlesex, and the University hospitals, for contributing £3000 to the expense of certain wards during three years, on condition that homœopathic treatment should be adopted for the patients, and a public "trial" made of homœopathy. He does not understand that this is a bribe offered to lull the consciences of men who refuse to tamper with the lives of the patients entrusted to their care, but laments the obstinacy which rejects his dangerous liberality.

